# Liver X receptors alpha gene (*NR1H3*) promoter polymorphisms are associated with systemic lupus erythematosus in Koreans

**DOI:** 10.1186/ar4563

**Published:** 2014-05-14

**Authors:** Ja-Young Jeon, Jin-Young Nam, Hyoun-Ah Kim, Yong-Beom Park, Sang-Cheol Bae, Chang-Hee Suh

**Affiliations:** 1 Department of Rheumatology and BK21 Division of Cell Transformation and Restoration, Ajou University School of Medicine, 164 Worldcup-ro, Yeongtong-gu, Suwon 443-380, Korea; 2 Division of Rheumatology, Department of Internal Medicine, Department of Medical Sciences, Yonsei University College of Medicine, Seoul, Korea; 3 Department of Rheumatology, Hanyang University Hospital for Rheumatic Diseases, Seoul, Korea

## Abstract

**Introduction:**

Liver X receptors are established sensors of lipid and cholesterol homeostasis. Recent studies have reported that these receptors are involved in the regulation of inflammation and immune responses. We attempted to identify single nucleotide polymorphisms (SNPs) of the *NR1H3* gene associated with the susceptibility to systemic lupus erythematosus (SLE).

**Methods:**

SNPs were genotyped using SNaPSHOT assay in 300 Korean patients with SLE and 217 normal controls (NC), and in replication samples (160 SLE patients and 143 NC). Also, the functional effects of *NR1H3* gene promoter polymorphisms were analyzed using a luciferase assay, real-time polymerase chain reaction, B cell proliferation assay and an electrophoretic mobility shift assay.

**Results:**

We identified five polymorphisms: -1851 T > C (rs3758673), -1830 T > C (rs3758674), -1003 G > A (new), -840 C > A (rs61896015) and -115 G > A (rs12221497). There was a significant and reproducible difference in the -1830 T > C, -1003 G > A and -115 G > A polymorphisms between the SLE and the NC. Luciferase activity of the structure containing -1830 C was less enhanced compared to the structure containing -1830 T in basal, GW3965 and T0901317 treated Hep3B cells (*P* = 0.009, *P* = 0.034 and *P* <0.001, respectively). Proliferation of the -1830 TC type was increased compared to the -1830 TT type in basal, GW3965 and T0901317 treated B cells from SLE patients (*P* = 0.011, *P* = 0.040 and *P* = 0.017, respectively). Transcription factor GATA-3 preferentially bound the -1830 T allele in the promoter.

**Conclusions:**

*NR1H3* genetic polymorphisms may be associated with disease susceptibility and clinical manifestations of SLE. Specifically, -1830 T > C polymorphism within *NR1H3* promoter region may be involved in regulation of *NR1H3* expression.

## Introduction

Systemic lupus erythematosus (SLE) is an autoimmune disease characterized by dysregulation of the immune system involving the hyperactivity of T and B cells, elevated production of pathogenic autoantibodies, complement activation, and the formation of immune complexes causing multiorgan damage by deposition in host tissue [1]. Although the exact pathogenesis of SLE remains elusive, extremely complicated and multifactorial interactions between genetic and environmental factors are thought to contribute to the development of the disease [2]. Genetic variation of various genes may lead to different inflammation, immune responses and susceptibility to SLE [3]. Several genetic association studies have been performed in patients with SLE and various genes encoding proteins with regulatory or adaptive functions in the immune system have been considered as candidates [4,5]. Well-established risk factors include alleles in the major histocompatibility complex, interferon regulatory factor 5, integrin alpha M, signal transducer and activator of transcription 4 and B lymphoid tyrosine kinase in genome-wide association studies in SLE [6,7]. Many susceptibility genes fall into key pathways that are consistent with previous studies implicating immune complexes, host immune signal transduction and interferon pathways in the pathogenesis of SLE [6,8].

Liver X receptor (LXR) alpha (*NR1H3*) and beta (*NR1H2*) can influence macrophage biology by modulation of lipid metabolism and by effects on innate immunity. The release of cytokines including interleukin (IL)-1, IL-6 and tumor necrosis factor-α from macrophages results in recruitment of monocytes and cross-talk with T cells, perpetuates cellular activation and further promotes atherosclerotic lesion development [9]. The anti-inflammatory effect of LXRs was first described in a study that demonstrated that LXR activation attenuated *Escherichia coli*- or lipopolysaccharide (LPS)-induced expression of pro-inflammatory molecules including IL-6, inflammatory nitric oxide synthase and cyclooxygenase-2 in macrophages from wild type mice, but not LXR null mice [10]. LXR is reportedly essential for macrophage survival and clearance of invading bacteria in protective immune responses [10,11], whereas LXR activation also inhibits lymphocyte proliferation [12]. A recent study found that LXRs mediate the regulation of Th17 cell differentiation and autoimmunity [13]. However, the possible association between *LXRs* genetic polymorphisms and SLE has not been addressed.

In this study, we attempted to identify polymorphisms of the *NR1H3* and *NR1H2* genes associated with susceptibility to SLE in Koreans and to elucidate the functional effect of these polymorphisms.

## Methods

### Study subjects

Three hundred SLE patients and 217 normal controls (NC) were enrolled from Ajou University Hospital in Suwon, Korea. All patients satisfied at least four of the 1982 revised American College of Rheumatology (ACR) criteria for SLE [14]. The patients’ medical histories were reviewed from the onset of disease until admission to the study. Clinical features of the disease, as defined by ACR criteria, were recorded in standardized questionnaires. Information about the medical history, clinical symptoms and physical examination were registered by a rheumatologist in a database when blood sampling was done. For each patient, blood cell count, routine chemistry, urinalysis, C-reactive protein and anti-dsDNA antibody were examined. Anti-dsDNA antibody was measured by radioimmunoassay using a commercial kit (Trinity Biotech, Bray, Ireland). Clinical manifestations including oral ulcer, arthritis, serositis, rash, nephritis, leukopenia (<4 × 10^3^ cells/μL), lymphopenia (<1 × 10^3^ cells/μL) and thrombocytopenia (<100 × 10^3^ cells/μL), anti-dsDNA antibody (>7.0 IU/ml) and anti-cardiolipin antibody (either or both immunoglobulin G (IgG) >20 GPL-U/mL and IgM positive >20 MPL-U/mL) were defined by positive involvement when it was positive at least once during the disease duration. The NCs were chosen from the general population using a screening questionnaire, which had to indicate no history of rheumatic diseases or autoimmune disorders. Also, replication samples were collected from other SLE patients (n = 160) and NC (n = 143). All the subjects who participated in this study were ethnically Korean. The study was approved by the Institutional Review Board of Ajou University Hospital and all subjects gave their informed consent.

### Identification and genotyping of SNPs

Fifty SLE patients and 50 NC Korean volunteers were used for SNP identification. Genomic DNA was extracted from whole blood using the QuickGene DNA whole blood kit S (Fujifilm Life Science, Tokyo, Japan). The *NR1H3* gene located between the promoter region and intron 2 region was amplified by polymerase chain reaction (PCR) with Amfisure PCR Master Mix (GenDEPOT, Barker, TX, USA). The *NR1H2* gene located in the promoter region and between the exon 10 region and the 3’ untranslated region were amplified by PCR. We identified possible polymorphisms in the *NR1H3* and *NR1H2* genes that were screened by direct sequencing (Bionics, Seoul, Korea). A minor allele frequency ≥5% was considered to indicate a SNP. Additionally, SNP genotyping was performed using the SNaPSHOT ddNTP primer extension kit (Applied Biosystems, Foster City, CA, USA) for SLE patients (n = 250) and NC (n = 167) and replication samples (160 SLE patients and 143 NC).

### Preparation of promoter constructs

Six reporter structures composed of the *NR1H3* -1830 T > C, -1003 G > A and -115 G > A sequence carrying each allele and the luciferase reporter gene were transfected into the Hep3B cell line. A 500 bp-sized fragment (from -2121 to -1622) of the *NR1H3* gene was PCR-amplified using either -1830 T homozygous or -1830 C homozygous genomic DNA as a template and the following primers: forward primer: 5′- CGGCGG**GGTACC**ACATCT ATGCCAGCCCTGTTTCAG -3′; the bold characters represent the KpnI site, reverse primer: 5′- CCGCCG**CTCGAG**ACTGAGCCCCAGCGGCTTTC -3′; the bold characters denote the XhoI site. A 500 bp-sized fragment (from -1266 to -767) of the *NR1H3* gene was PCR amplified using either -1003 G homozygous or -1003 A homozygous genomic DNA as a template and the following primers: forward primer: 5′- CTATCGATA**GGTACC**CTCCCCTCAGCCTTTCCCCA -3′; reverse primer: 5′- ATCGCAGAT**CTCGAG**TCCCCCTCACTC CAACACTGAG -3′. A 500 bp-sized fragment (from -374 to +126) of the *NR1H3* gene was PCR amplified using either -115 G homozygous or -115 A homozygous genomic DNA as a template and the following primers: forward primer: 5′- CTATCGATA**GGTACC**GTTTTG ACCTCAGAGGGATATTAT -3′; reverse primer: 5′- ATCGCAGAT**CTCGAG**CAGGTAATGAAGGAGGCTGA -3′. Each PCR product was subcloned separately into the KpnI–XhoI site of the pGL3-Basic luciferase reporter vector (Promega, Madison, WI, USA).

### Transfection and luciferase reporter assays

Hep3B cells (hepatocellular carcinoma cell line #58064; Korean Cell Line Bank (KCLB), Seoul, Korea) were cultured in Roswell Park Memorial Institute (RPMI) 1640 (Invitrogen, Carlsbad, CA, USA) and COS-7 cells (African Green Monkey kidney cell line #21651, KCLB) were cultured in high-glucose (D)MEM (Hyclone, Logan, UT, USA) supplemented with 10% heat-inactivated fetal bovine serum (FBS) at 37°C in a 5% CO_2_ incubator. Hep3B and COS-7 cells were transfected by using FuGENE6 (Roche, Mannheim, Germany) according to the manufacturer’s instructions. Reporter plasmid DNA of *NR1H3* 0.5 μg and pSV-β-galactosidase plasmid DNA (Promega) 0.5 μg were resuspended and further gently mixed with 1.5 μL of FuGENE6 reagents described previously [15]. The relative transcriptional activity of each construct was expressed as the ratio of luciferase activity to β-galactosidase activity using a luminometer, the LUMIstar OPTIMA (BMG LABTECH, Ortenberg, Germany). The transfections and luciferase assays were conducted in triplicate, and the experiments were repeated at least three times with different cell preparations as described previously [15].

### Pharmacological treatment and reporter gene assay

Hep3B and COS-7 cells were transfected with *NR1H3* promoter pGL3-basic constructs using FuGENE6 (Promega) according to the manufacturer’s instructions. After incubation for six hours, the medium was replenished with 500 μL of fresh medium with 20% FBS, and the cells were incubated a further 18 hours at 37°C in a 5% CO2 incubator. Twenty four hours after transfection, cells were treated with either 200 ng/mL LPS (Sigma-Aldrich, St. Louis, MO, USA), 3 μmol/L 3-[3-[N-(2-chloro-3-trifluoro methylbenzyl)-(2,2-diphenylethyl)amino]propyloxy]phenylacetic acid hydrochloride (hydrochloride GW3965; GW3965, Sigma-Aldrich) or 5 μmol/L N-(2,2,2-trifluoroethyl)-N-[4-[2,2,2-trifluoro-1-hydroxy-1-(trifluoromethyl)ethyl]phenyl]-benzenesulfonamide (sulfonamide T0901317; T0901317, Cayman, Ann Arbor, MI, USA) for 24 hours. These concentrations and time were chosen based on previous work [16].

### Primary culture and Epstein-Barr virus transformation of lymphocytes

The peripheral blood mononuclear cells were separated from peripheral blood using Histopaque (Sigma-Aldrich) and counted to provide 1 × 10^6^ cells/ml. Then, the prepared Epstein-Barr virus (EBV; B95-8 cell line) was added. After 24 hours incubation in a CO2 incubator, 0.5 μg/ml cyclosporine A was added and the cells cultured for three weeks. After one week, cell line formation was confirmed. After two weeks, adequate transformation was confirmed by phase microscopy (until medium begins to turn orange/yellow and small clumps of cells become visible). The media change step was repeated until the total cell number exceeded 5 × 10^6^ cells/ml. Finally, the transformed cells were retrieved and preserved at -80°C in a deep freezer.

### Total RNA extraction and quantitative real-time RT-PCR

The EBV-transformed B cell line was cultured in RPMI 1640 supplemented with 10% heat-inactivated FBS at 37°C in a 5% CO2 incubator. Total RNA was extracted from B cells using a RNA mini extraction kit (Inclone, Seoul, Korea) according to the manufacturer’s suggested protocol. Total RNA (2 μg) was converted to cDNA by a GoScript reverse transcription system kit (Promega) according to the manufacturer’s suggested protocol. After annealing at 25°C for five minutes, extension at 42°C for one hour, inactive reverse transcriptase at 70°C for 15 minutes, the product was stored at -20°C until used.

The reverse transcription PCR (RT-PCR) reaction was performed by PCR with amfisure PCR Master Mix (GenDEPOT) under the following conditions: hot start at 94°C for four minutes followed by 37 cycles of 95°C for one minute, 58°C for one minute, and 72°C for thirty seconds with a final extension at 72°C for ten minutes. *NR1H3* was amplified with the following primers: forward primer: 5′- AGGGCTGCAAGGGATTCTTCC -3′, reverse primer: 5′- TCTGACAGCACACACTCCTCCC -3′. Samples were subjected to parallel amplification of the constitutively expressed, housekeeping gene, human β-actin using the following primers: forward primer: 5′- TCCTTCTGCATCCTGTCGGC -3′, reverse primer: 5′- CAAGAGATGGCCACGGCTGC -3′.

The real-time PCR reaction was performed by a Rotor-Gene SYBR Green PCR kit (Qiagen, Valencia, CA, USA) with the same primers of RT-PCR under the following conditions: heating to 95°C for five minutes, and 40 cycles of 95°C for five seconds, 58°C for ten seconds, and 72°C for thirty seconds.

### B cell proliferation assay

B cells were seeded into 96-well plates at a density of 1 × 10^5^ cells. After incubation for 24 hours, cells were treated with either 200 ng/mL LPS (Sigma-Aldrich), 3 μmol/L GW3965 (Sigma-Aldrich) or 5 μmol/L T0901317 (Cayman) for 24 hours. The concentrations and time were chosen based on previous data. After incubation for 24 hours, cell viability was assessed using the CellTiter 96 AQueous One Solution Cell Proliferation Assay (Promega). This is a colorimetric assay containing a tetrazolium compound. When the tetrazolium is reduced, it produces a colored formazan product that is soluble in cell culture medium and is maximally absorbed at 490 nm. Assays were conducted in triplicate, and the experiments were repeated at least three times.

### Nuclear extract preparation and electrophoretic mobility shift assay

Hep3B cells were washed with ice-cold phosphate-buffered saline before being suspended in ice-cold hypoosmotic buffer comprised of 10 mM HEPES/KOH (pH 7.9), 2 mM MgCl2, 0.1 mM ethylenediaminetetraactic acid (EDTA), 10 mM KCl, 1 mM dithiothreitol (DTT), 1 mM phenylmethanesulfonylfluoride (PMSF), 1 mM NaVO3, 10 mM NaF and complete protease inhibitor cocktail (Roche, Basel, Switzerland). The cells were incubated on ice for 10 minutes and for an additional 15 minutes in the presence of 0.2% Nonidet P-40. After centrifugation at 15,000 × g for 30 seconds, the pellet was resuspended in a cold saline buffer that contained 50 Mm HEPES/KOH (pH 7.9), 50 mM KCl, 300 mM NaCl, 0.1 mM EDTA, 10% (v/v) glycerol, 1 mM DTT, 1 mM PMSF and protease inhibitors. The mixture was incubated on ice for 30 minutes. After centrifugation at 15,000 × g for 30 minutes at 4°C, the supernatant containing the nuclear proteins was stored at -80°C until use. The following double-stranded oligonucleotides were used as probes for the electrophoretic mobility shift assay (EMSA): for *NR1H3* -1830 T > C 5′ - AGATTTCCTA[t/c]CAAAGGCTCT - 3′, CdxA as a nonspecific competitor, 5′ - AGATCTGGTACCATTTAAGCCCTCGAGATCTA - 3′ and for GATA-3 as a specific competitor, 5′ - GTTATTTATCTCTTAGTTGTAGTTATTTATCTCTTGTTGTA - 3′. The double-stranded oligonucleotides were annealed by incubation at 95°C for five minutes followed by gradual cooling to room temperature. The double-stranded DNA probes were radiolabeled at their 5′-ends with DNA polymerase I (Klenow fragment; Promega) and [α-32P]CTP. The reaction mixtures (10 μl), which contained 5 μg of nuclear extract, 10 mM HEPES (pH 7.9), 250 ng poly(dI:dC), 0.005 mM MgCl_2_, 75 mM NaCl, 5 mM DTT, 1 mM NaNO_3_, 10 mM NaF, 1 mM EDTA, 400 ng bovine serum albumin and 50% glycerol, were preincubated on ice for 20 minutes, and then incubated for 20 minutes on ice in the presence of 0.2 pmol of radiolabeled probe. The reaction mixtures were then subjected to electrophoresis on a native 6% polyacrylamide gel. For competition experiments, unlabeled blunt-ended competitor oligonucleotides were added to the binding reaction mixtures before the addition of the radiolabeled oligonucleotide probes. The gels were dried and radioactivity was detected using a FLA3000 scanner (Fuji Photo Film, Tokyo, Japan).

### Statistical analyses

The genotype frequency was tested for significant departure from Hardy-Weinberg equilibrium at each SNP by chi-square analysis. Differences in genotype frequency between the cases and controls were tested by the chi-square test and calculation of the odds ratio (OR) and the 95% confidence interval (CI). Three logistic regression models (codominant, dominant and recessive) were used to analyze the SNP after controlling for age and sex as covariates. Differences in the mean value of the phenotypic characteristics between groups were compared by an analysis of variance (ANOVA) test and a t-test. *P* values <0.05 were considered to be significant. Haplotypes were analyzed using Haploview version 4.2 based on the EM algorithm [17]. Linkage disequilibrium between loci was measured using the absolute value of Lewontin’s /D′/ and *r*^*2*^[18]. Statistical analyses were conducted using the SPSS version 12.0 software (SPSS, Chicago, IL, USA).

## Results

### Clinical characteristics of the study subjects

The mean age of the SLE patients was 30.6 ± 9.2 years and 89.0% were female. The mean age of the NC was 29.8 ± 5.8 years and 88.5% were female. Clinical features of SLE patients are as follows with decreasing frequency: arthritis (67.3%), oral ulcer (49.3%), rash (39.0%), nephritis (26.3%) and serositis (13.3%). In replication samples, the mean age of the SLE patients was 42.3 ± 13.4 years and 81.3% were female. The mean age of the NC was 41.7 ± 9.7 years and 81.8% were female (Additional file 1).

### Genotype and haplotype frequencies

The allele and genotype frequencies of the *NR1H3* polymorphisms are presented in Table 1. Genotype distributions of all the polymorphisms satisfied the Hardy–Weinberg proportions in the NC (*P* >0.05). Based on an allele frequency ≥5%, five SNPs of the *NR1H3* gene were identified; -1851 T > C (rs3758673) and -1830 T > C (rs3758674) in the promoter region, and -1003 G > A (new), -840 C > A (rs61896015) and -115 G > A (rs12221497) in the intron 1 region.

**Table 1:** **The genotype and allele frequencies of polymorphisms in the *NR1H3 *** **gene**

**Loci**	**Genotype**	**Our**				**Replication**				**Combine**			
		**SLE (n = 300)**	**NC (n = 217)**	***P *** **value**	**OR (95% CI)**	**SLE (n = 160)**	**NC (n = 143)**	***P *** **value**	**OR (95% CI)**	**SLE (n = 460)**	**NC (n = 360)**	***P *** **value**	**OR (95% CI)**
-1851 T > C	TT	172 (57.3%)	115 (53.0%)	co : 0.281	1.171 (0.879 ~ 1.559)	93 (58.1%)	78 (54.5%)	co : 0.578	1.107 (0.577 ~ 1.851)	265 (57.6%)	193 (53.6%)	co : 0.215	1.151 (0.921 ~ 1.438)
	TC	112 (37.3%)	57 (40.1%)	do : 0.507	1.132 (0.786 ~ 1.630)	55 (34.4%)	53 (37.1%)	do : 0.816	1.051 (0.689 ~ 1.603)	167 (36.3%)	140 (38.9%)	do : 0.425	1.118 (0.723 ~ 2.163)
	CC	16 (5.3%)	15 (6.9%)	re : 0.316	1.094 (0.918 ~ 1.305)	12 (7.5%)	12 (8.4%)	re : 0.557	1.071 (0.852 ~ 1.347)	28 (6.1%)	27 (7.5%)	re : 0.252	1.085 (0.944 ~ 1.246)
	q.	0.240	0.270	0.285	1.080 (0.938 ~ 1.245)	0.250	0.270	0.567	1.055 (0.878 ~ 1.267)	0.242	0.269	0.212	1.074 (0.960 ~ 1.201)
	HWE			0.790				0.487				0.817	
-1830 T > C	TT	238 (79.3%)	195 (89.9%)	co : 0.001	0.431 (0.260 ~ 0.714)	131 (81.9%)	129 (90.2%)	co : 0.034	0.483 (0.246 ~ 0.948)	369 (80.2%)	324 (90.0%)	co : <0.001	0.447 (0.299 ~ 0.668)
	TC	58 (19.3%)	22 (10.1%)	do : 0.999	0.000 (0.000 ~ NA)	28 (17.5%)	14 (9.8%)	do : 1.000	0.000 (0.000 ~ NA)	86 (18.7%)	36 (10.0%)	do : 0.999	0.000 (0.000 ~ NA)
	CC	4 (1.3%)	0 (0.0%)	re : 0.002	0.658 (0.507 ~ 0.855)	1 (0.6%)	0 (0.00%)	re : 0.041	0.698 (0.495 ~ 0.985)	5 (1.1%)	0 (0.0%)	re : <0.001	0.671 (0.546 ~ 0.826)
	q.	0.110	0.051	0.001	0.658 (0.512 ~ 0.512)	0.090	0.050	0.038	0.706 (0.508 ~ 0.981)	0.104	0.05	<0.001	0.672 (0.552 ~ 0.820)
	HWE			0.432				0.538				0.318	
-1003G > A	GG	250 (83.3%)	201 (92.6%)	co : 0.002	0.396 (0.223 ~ 0.705)	139 (86.9%)	131 (91.6%)	co : 0.194	0.606 (0.285 ~ 1.289)	389 (84.6%)	332 (92.2%)	co : 0.001	0.460 (0.293 ~ 0.721)
	GA	47 (15.7%)	16 (7.4%)	do : 0.999	0.000 (0.000 ~ NA)	21 (13.1%)	12 (8.4%)	do : NA	NA	63 (14.8%)	28 (7.8%)	do : 0.999	0.000 (0.000 ~ NA)
	AA	3 (1.0%)	0 (0.0%)	re : 0.002	0.629 (0.468 ~ 0.847)	0 (0.0%)	0 (0.0%)	re : 0.194	0.779 (0.534 ~ 1.135)	3 (0.7%)	0 (0.0%)	re : 0.001	0.680 (0.540 ~ 0.856)
	q.	0.088	0.037	0.001	0.626 (0.470 ~ 0.835)	0.070	0.040	0.209	0.791 (0.549 ~ 1.140)	0.080	0.039	0.001	0.680 (0.544 ~ 0.850)
	HWE			0.573				0.600				0.443	
-840 C > A and -115G > A	GG	235 (78.3%)	195 (89.9%)	co : <0.001	0.408 (0.246 ~ 0.675)	131 (81.88%)	129 (90.21%)	co : 0.034	0.483 (0.246 ~ 0.948)	366 (79.6%)	324 (90.0%)	co : <0.001	0.430 (0.288 ~ 0.642)
	GA	61 (20.3%)	22 (10.1%)	do : 0.999	0.000 (0.000 ~ NA)	28 (17.5%)	14 (9.79%)	do : 1.000	0.000 (0.000 ~ NA)	89 (19.3%)	36 (10.0%)	do : 0.999	0.000 (0.000 ~ NA)
	AA	4 (1.3%)	0 (0.0%)	re : 0.001	0.639 (0.493 ~ 0.829)	1 (0.63%)	0 (0.00%)	re : 0.041	0.698 (0.495 ~ 0.985)	5 (1.1%)	0 (0.0%)	re : <0.001	0.658 (0.535 ~ 0.808)
	q.	0.115	0.051	<0.001	0.642 (0.501 ~ 0.823)	0.090	0.050	0.038	0.706 (0.508 ~ 0.981)	0.108	0.050	<0.001	0.661 (0.542 ~ 0.805)
	HWE			0.432				0.538				0.318	

Each *P* value was calculated with co-dominant (co), dominant (do) and recessive (re) models. Logistic regression analysis was applied to control for age and sex as covariables. Each q row’s *P* value was analyzed by the Chi square test. Two genetic polymorphisms of the NR1H3 gene, -840 C > A and -115 G > A, were in complete linkage disequilibrium. CI, confidence interval; HWE, Hardy–Weinberg equilibrium; NA, not applicable; OR, odds ratio; q., minor allele frequency.

In the -1830 T > C polymorphism, the genotype frequency of the minor allele was significantly higher in the SLE patients when compared to the NC (*P* = 0.001 for the co-dominant model, *P* = 0.002 for the recessive model). Also, the minor allele of the -1003 G > A polymorphism was significantly more frequent in the SLE patients than in the NC (*P* = 0.002 for the co-dominant model, *P* = 0.002 for the recessive model). In addition, the SLE patients had the minor allele of the -115 G > A polymorphism more frequently (*P* = <0.001 for the co-dominant model, *P* = 0.001 for the recessive model). However, screening of the *NR1H2* gene for genetic variation did not reveal differences between the SLE and the NC (data not shown).

In the replication samples, the minor allele of the -1830 T > C and -115 G > A polymorphisms were significantly more frequent in the SLE patients than in the NC (*P* = 0.034 for the co-dominant model, *P* = 0.041 for the recessive model). Moreover, in combined samples, the minor allele of the -1830 T > C and -115 G > A polymorphisms were significantly more frequent in the SLE than in the NC (*P* = <0.001 for the co-dominant model, *P* = <0.001 for the recessive model). Although there was no difference in the -1003 G > A polymorphism in replication samples, the minor allele of the -1003 G > A polymorphism was significantly more frequent in the combined SLE patients than in the combined NC (*P* = 0.001 for the co-dominant model, *P* = 0.001 for the recessive model).

Linkage disequilibrium between SNPs and locus by locus was examined. Two genetic polymorphisms of the *NR1H3* gene, -840 C > A and -115 G > A, were in complete linkage disequilibrium. Therefore, -840 C > A was excluded in the haplotype analysis. Three common haplotypes for four polymorphisms were constructed using the Haploview software: HT1 [TTGG], HT2 [CTGG] and HT3 [TCAA]. There was a significant difference between SLE and NC in the observed haplotype HT1 [TTGG] (*P* = 0.033 for the co-dominant model, *P* = 0.012 for the recessive model) and HT3 [TCAA] (*P* = 0.008 for the co-dominant model, *P* = 0.009 for the dominant model) (Table 2).

**Table 2:** **The haplotype frequencies of *NR1H3 *** **gene**

**Haplotype**		**SLE**	**NC**	**SLE versus NC**	
		**(n = 300)**	**(n = 217)**	***P *** **value**	**OR (95% CI)**
HT1	+/+	109 (36.3%)	103 (47.5%)	co : 0.033	0.727 (0.542 ~ 0.974)
[TTGG]	+/-	169 (56.3%)	99 (45.6%)	do : 0.808	0.956 (0.681 ~ 1.349)
	-/-	22 (7.3%)	15 (6.9%)	re : 0.012	0.796 (0.666 ~ 0.951)
HT2	+/+	16 (5.3%)	15 (6.9%)	co : 0.255	0.847 (0.636 ~ 1.128)
[CTGG]	+/-	111 (37.0%)	87 (40.1%)	do : 0.280	0.908 (0.761 ~ 1.082)
	-/-	173 (57.7%)	115 (53.0%)	re : 0.507	0.884 (0.614 ~ 1.273)
HT3	+/+	2 (0.7%)	0 (0.0%)	co : 0.008	2.739 (1.304 ~ 5.751)
[TCGG]	+/-	30 (10.0%)	9 (4.1%)	do : 0.009	1.662 (1.134 ~ 2.435)
	-/-	268 (89.3%)	208 (95.9%)	re : 0.999	36085.247 (0.000 ~ NA)
HT others	+/+	3 (1.0%)	0 (0.0%)	co : <0.001	8.375 (2.557 ~ 27.431)
	+/-	30 (10.0%)	3 (1.4%)	do : <0.001	2.984 (1.641 ~ 5.427)
	-/-	267 (89.0%)	214 (98.6%)	re : 0.999	32475.263 (0.000 ~ NA)

Haplotypes (HT) were analyzed using Haploview version 4.1 based on the EM algorithm. Each *P* value was calculated with co-dominant(co), dominant(do), and recessive(re) models. Logistic regression analysis was applied to control for age and sex as covariables. Two genetic polymorphisms of the *NR1H3* gene, -840 C > A and -115 G > A, were in complete linkage disequilibrium. Therefore, -840 C > A was excluded in the haplotype analysis. CI, confidence interval; NA, not applicable; OR, odds ratio.

### Associations between SLE phenotype and SNPs

The clinical characteristics according to genotype and haplotype of *NR1H3* gene are summarized in Table 3. In the -1830 T > C polymorphism, arthritis was significantly more common in the SLE patients with the -1830 C allele (*P* = 0.005). Also, arthritis (*P* = 0.006), anti-dsDNA (*P* = 0.041) and triglyceride (*P* = 0.011) were more common in the SLE patients with the -1003 A allele than the -1003 G allele, but oral ulcer (*P* = 0.039) and central nervous system (CNS) involvement (*P* = 0.015) were less common in the -1003 A allele. In addition, arthritis (*P* <0.001) was more common in the SLE patients with the -115 A allele than -115 C allele, but oral ulcer (*P* = 0.024) was less common in the -115 A allele (Additional file 2).

**Table 3:** **Comparison of the clinical characteristics according to the genotype and haplotype of *NR1H3 *** **gene in SLE**

**Characteristics**	**-1830 T > C**			**-1003G > A**			**-115G > A**		
	**TT**	**CT,CC**	***P***	**GG**	**GA,AA**	***P***	**GG**	**GA,AA**	***P***
	**n = 238 (79.3%)**	**n = 62 (20.7%)**	**value**	**n = 250 (83.3%)**	**n = 50 (6.7%)**	**value**	**n = 235 (78.3%)**	**n = 65 (21.7%)**	**value**
Oral ulcer^a^	122 (51.3%)	26 (41.9%)	0.191	130 (52.0%)	18 (36.0%)	0.039	124 (52.8%)	24 (36.9%)	0.024
Arthritis^a^	151 (63.4%)	51 (82.3%)	0.005	160 (64.0%)	42 (84.0%)	0.006	146 (62.1%)	56 (86.2%)	<0.001
Anti-ds DNA^a^	156 (65.5%)	47 (75.8%)	0.124	163 (65.2%)	40 (80.0%)	0.041	153 (65.1%)	50 (76.9%)	0.071
CNS involvement^a^	19 (8.0%)	3 (4.8%)	0.295	22 (8.8%)	0 (0.0%)	0.015	20 (8.5%)	2 (3.1%)	0.106
Triglyceride^b^	97.8 ± 64.1	112.8 ± 67.1	0.108	95.6 ± 61.5	126.9 ± 74.9	0.007	95.3 ± 62.1	120.9 ± 71.2	0.011
**Characteristics**	**HT1 [TTGG]**			**HT2 [CTGG]**			**HT3 [TCGG]**		
	**+/+**	**+/-, -/-**	***P***	**+/+**	**+/-, -/-**	***P***	**+/+, +/-**	**-/-**	***P***
	**n = 63 (41.7%)**	**n = 88 (58.3%)**	**value**	**n = 24 (15.9%)**	**n = 127 (84.1%)**	**value**	**n = 30 (19.8%)**	**n = 121 (80.1%)**	**value**
Arthritis^a^	62 (56.9%)	140 (73.3%)	0.004	9 (56.3%)	193 (68.0%)	0.331	29 (90.6%)	173 (64.6%)	0.003
Leukopenia^a^	61 (56.0%)	112 (58.6%)	0.652	11 (68.8%)	162 (57.0%)	0.356	20 (62.5%)	153 (57.1%)	0.558
Lymphopenia^a^	100 (91.7%)	172 (90.1%)	0.628	12 (75.0%)	260 (91.5%)	0.027	30 (93.8%)	242 (90.3%)	0.526

^a^This value is presented as the number of patients positive for the feature or antibody. ^b^This value was presented as the means ± SD. Leukopenia is defined as a leukocyte count that is less than 4 × 10^3^ cells/μL. Lymphopenia is defined as the lymphocyte count that is less than 1 × 10^3^ cells/μL. Logistic regression analysis was applied to control for age and sex as covariables. No association with SLE phenotypes was observed when the other SNPs were evaluated. No association with SLE phenotypes was observed when the other haplotypes were evaluated. HT, haplotype; SLE, systemic lupus erythematosus; SNP, single nucleotide polymorphism.

The frequency of arthritis was significantly lower in patients who had haplotype HT1 [TTGG] (recessive model, *P* = 0.004); however, arthritis was more common in patients with HT3 [TCGG] (dominant model, *P* = 0.003). The frequency of lymphopenia was significantly lower in patients who had haplotype HT2 [CTGG] (recessive model, *P* = 0.027) (Additional file 3).

### Transcriptional activity of the *NR1H3* gene according to the SNPs

**Figure 1:**
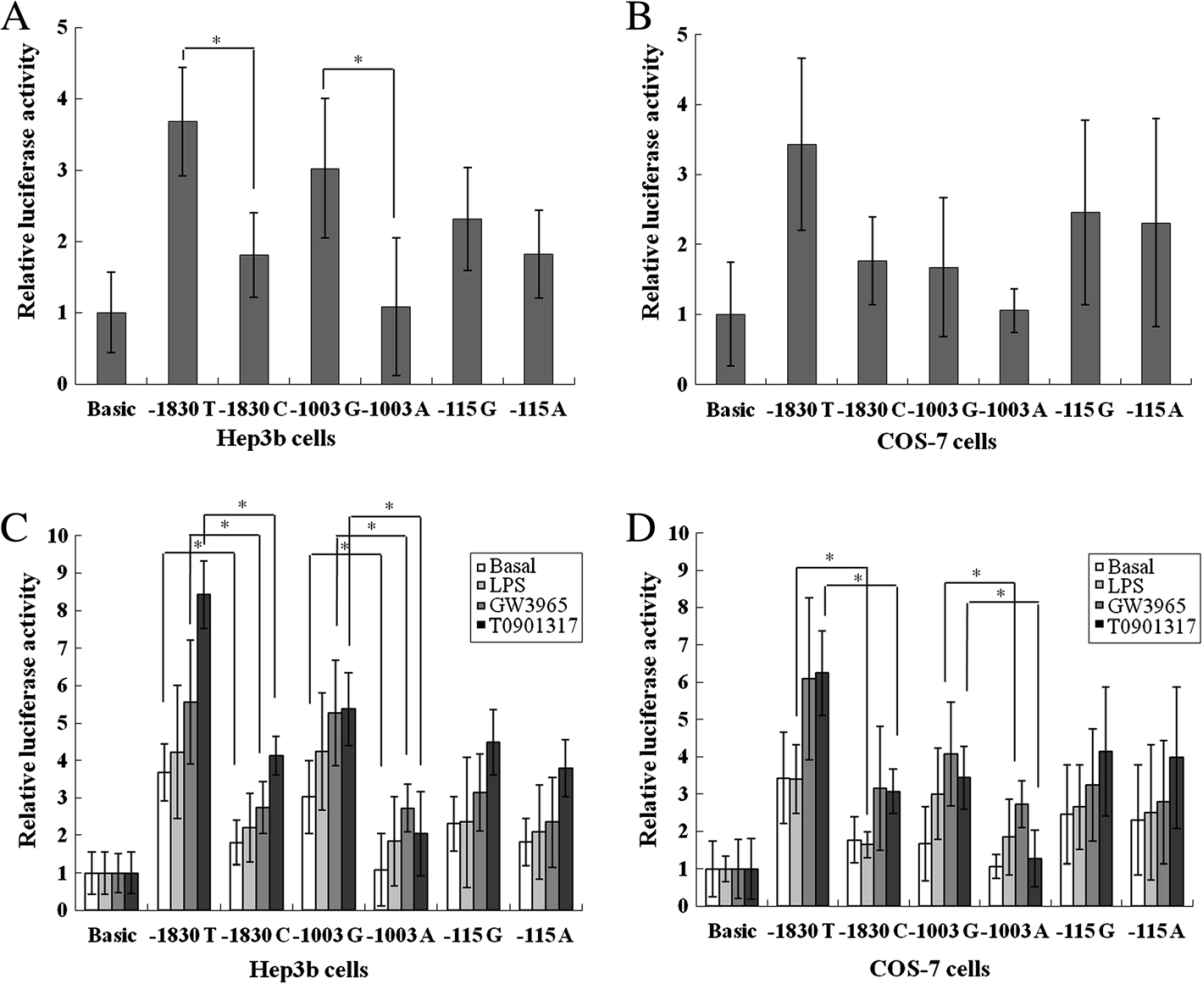
**Functional effect of the polymorphisms on transcriptional activity. (A, B)** Relative luciferase production (ratio of luciferase production driven by the reporter construct to that by the promoter-less control vector, pGL3-Basic) in Hep3B cells **(A)** and COS-7 cells **(B)**. **(C, D)** Relative luciferase production by stimulation with 200 ng/mL LPS, 3 μmol/L GW3965 and 5 μmol/L T0901317 in Hep3B cells **(C)** and COS-7 cells **(D)**. The *P* value was determined by a paired t test. *P <0.05. LPS, lipopolysaccharide.

To determine if the *NR1H3* -1830 T > C, -1003 G > A and -115 G > A polymorphisms are associated with altered promoter activity, six reporter structures composed of the promoter sequence carrying each allele and the luciferase reporter gene were transfected into the Hep3B cell line. The luciferase activity of the structure containing -1830 C was less enhanced when compared to that of the structure containing -1830 T (*P* = 0.009) (Figure 1A). The trend of enhanced promoter activity of the -1830 T structure is shown in different cell lines of the COS-7 cells (*P* = 0.052) (Figure 1B). The luciferase activity of the structure containing -1003 A was less enhanced when compared to that of the structure containing -1003 G (*P* = 0.030) (Figure 1A). The trend of enhanced transcriptional activity of the -1003 G structure is shown in different cell lines of the COS-7 cells (*P* = 0.061) (Figure 1B). There were no significant differences between -115 G and -115 A in the observed luciferase activity (Figure 1A, B).

### Pharmacological treatment and reporter gene assay

*NR1H3* transcriptional activity according to the stimulants (LPS and synthetic ligands, such as GW3965 and T0901317) was analyzed in Hep3B cells and COS-7 cells. The luciferase activity of -1830 C was less enhanced when compared to that of -1830 T in GW3965 and T0901317 treated Hep3B cells (*P* = 0.034 and *P* <0.001, respectively) (Figure 1C). The reduced enhancement of transcriptional activity of the -1830 C structure was reconfirmed in LPS- and T0901317-treated COS-7 cells (*P* = 0.023 and *P* = 0.006, respectively) (Figure 1D). The luciferase activity of -1003 A was less enhanced when compared to that of -1003 G in GW3965- and T0901317-treated Hep3B cells (*P* = 0.038 and *P* = 0.004, respectively) (Figure 1C). Less enhanced transcriptional activity of the -1003 A structure was replicated in GW3965- and T0901317-treated COS-7 cells (*P* = 0.029 and *P* = 0.009, respectively) (Figure 1D). However, there were no significant differences between -115 G and -115 A in the observed luciferase activity in Hep3B cells and COS-7 cells treated with LPS, GW3965 and T0901317 (Figure 1C, D).

### Nuclear extract preparation and electrophoretic mobility shift assay

**Figure 2:**
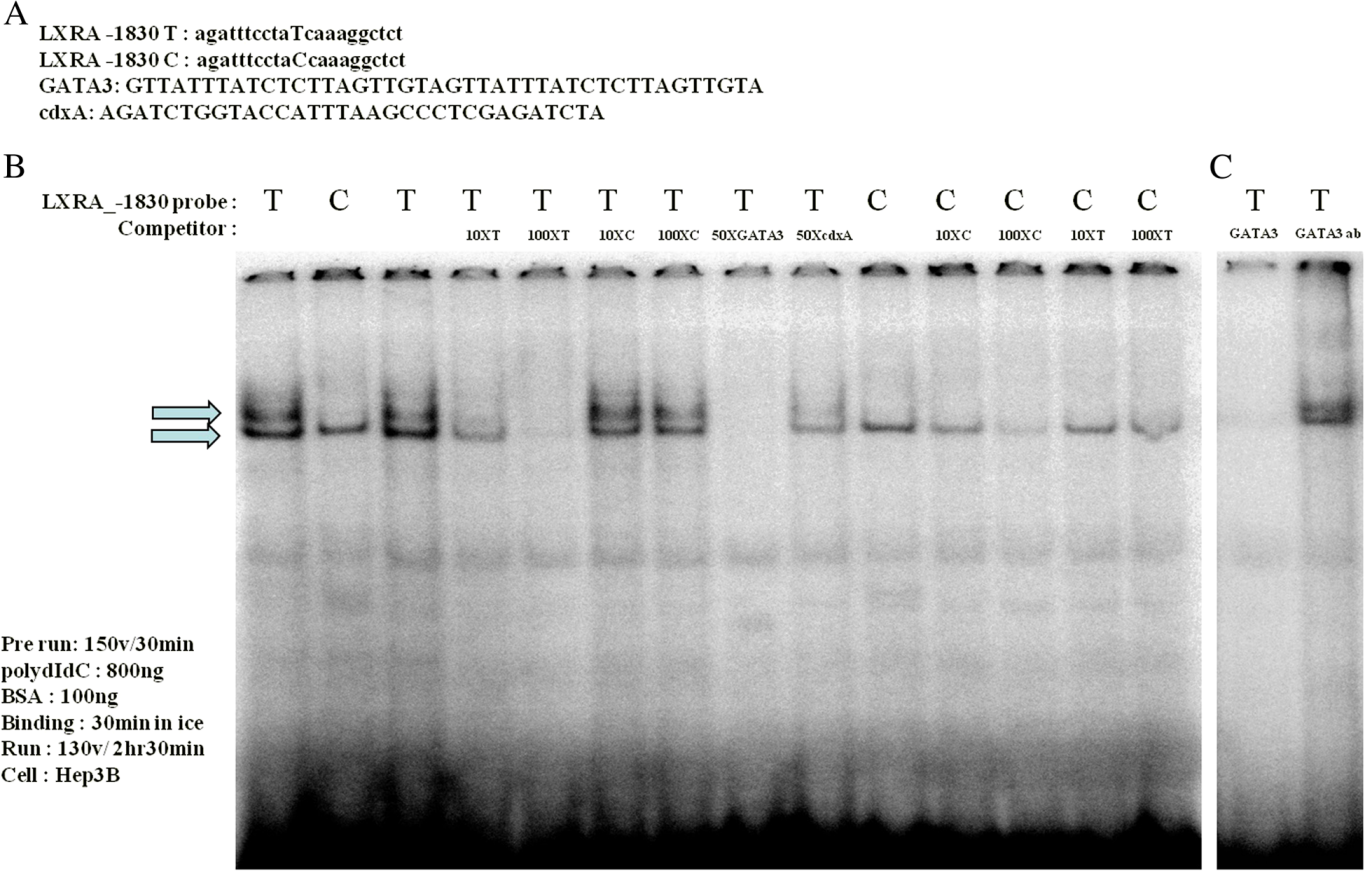
**Results of an EMSA for the -1830 T > C polymorphism using nuclear extracts obtained from Hep3B cells. (A)** Nucleotide sequences of oligonucleotides used as probes and competitors. **(B)** Differential binding of a nuclear protein to -1830 T > C alleles. **(C)** Identification of the transcription factor binding to 1830 T > C probe using competitive binding assay. EMSA, electrophoretic mobility shift assay.

EMSA using nuclear extracts prepared from Hep3B cells revealed a specific band with the -1830 T probe, but not with the -1830 C probe. The shifted band produced by the -1830 T probe was not visible in the presence of the nonlabeled -1830 T probe but remained visible in the presence of the nonlabeled -1830 C probe (Figure 2A). A survey of a database of transcription factors suggested that the -1830 T > C polymorphism generated a potential GATA-3 binding motif. To clarify the involvement of GATA-3, we performed a competition assay using GATA-3 and CdxA probes, and found that the shifted band corresponding to the -1830 T probe was completely competed for by the unlabeled GATA-3 probe, but not by the unlabeled CdxA probes (Figure 2B). The -1830 T specific band was also competed by anti-GATA-3 antibodies (Santa Cruz Biotechnology, Santa Cruz, CA, USA) but not supershifted (Figure 2C).

### *NR1H3* mRNA expression according to genotype

**Figure 3:**
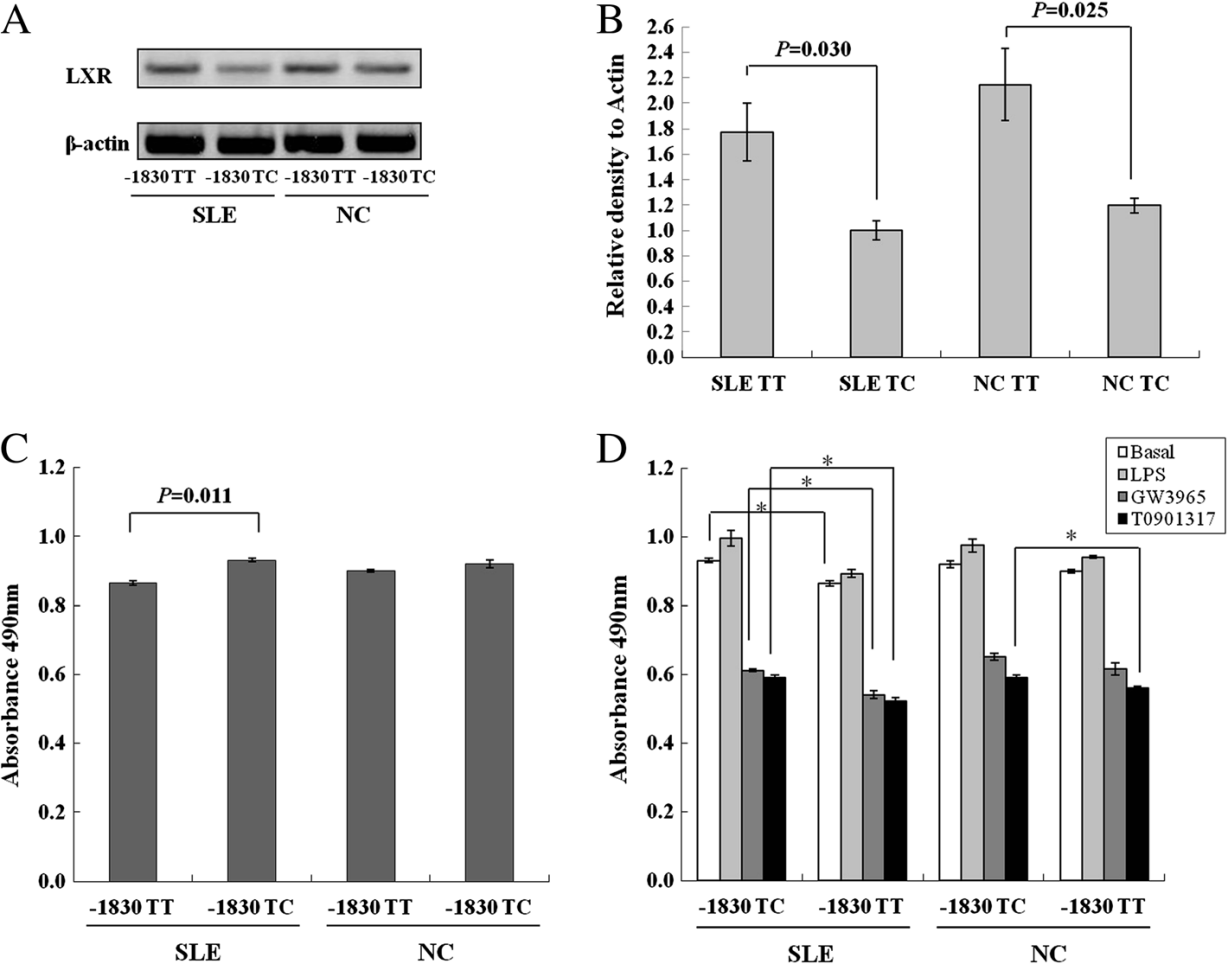
**Effect of the -1830 T > C polymorphism on *NR1H3 *mRNA expression and B cells proliferation assay. (A)** RT PCR products were separated by electrophoresis on 1% agarose gel and stained with EtBr. Gels were examined by ultraviolet illumination. **(B)** The mRNA levels were determined by quantitative-PCR normalized to β-actin mRNA expression. Each experiment was conducted in duplicate for each sample, and the results are expressed as mean ± SD for three independent experiments. The *P* value was determined by a paired t test. **(C)** The effect of the -1830 T > C polymorphism on the *NR1H3* gene on the B cells proliferation assay. This is a colorimetric assay containing a tetrazolium compound. When the tetrazolium is reduced, it produces a colored formazan product that is soluble in cell culture medium and maximally absorbed at 490 nm. Assays were conducted in triplicate, and the experiments were repeated at least three times. Values are mean ± SD; *P <0.05. **(D)** Effect of the -1830 T > C polymorphism on the *NR1H3* gene on B cells proliferation by stimulation with 200 ng/mL LPS, 3 μmol/L GW3965 and 5 μmol/L T0901317. Values are mean ± SD. The *P* value was determined by a paired t test. *P <0.05. LPS, lipopolysaccharide; SD, standard deviation.

B cell lines were screened to measure mRNA expression of *NR1H3* gene according to the genotypes using RT-PCR. As illustrated in Figure 3A, the -1830 TC type B cells displayed a lower *NR1H3* mRNA expression level than the -1830 TT type B cells in SLE. Also, the -1830 TC type B cells displayed a lower *NR1H3* mRNA expression level than the -1830 TT type B cells in NC. Moreover, the *NR1H3* expression level was significantly different between genotypes in real-time PCR. The -1830 TC type had an approximately 1.8-fold decrease compared to the -1830 TT type in SLE. Also, the -1830 TC type had an approximately 1.8-fold decrease compared to the -1830 TT type in NC (*P* = 0.03 and *P* = 0.025, respectively) (Figure 3B).

### Effect of LXR agonists on B cell proliferation according to the genotypes

B cell proliferation assay was performed to determine the effect of -1830 T > C polymorphism. Proliferation of -1830 TC type was increased compared to that of -1830 TT type in B cells from SLE patients (*P* = 0.011) (Figure 3C). Proliferation of the -1830 TC type was increased when compared to that of the -1830 TT type in GW3965- and T0901317-treated B cells from SLE patients (*P* = 0.040 and *P* = 0.017, respectively) (Figure 3D).

## Discussion

In the present study, we evaluated the associations of genetic polymorphisms of the *NR1H3* and *NR1H2* with SLE in a Korean population. There were five genetic polymorphisms of the *NR1H3* gene at -1851 T > C, -1830 T > C, -1003 G > A, -840 C > A and -115 G > A including one novel SNP (-1003 G > A) between the promoter region and the second intron region. Also, we found that the rare alleles of the -1830 T > C, -1003 G > A and -115 G > A polymorphisms were associated with a significantly higher disease susceptibility. In the haplotype analysis, there was a significant difference between SLE and NC in the observed haplotype HT1 [TTGG] and HT3 [TCAA]. Our results suggest that the human *NR1H3* gene plays an important role in the development of SLE.

Some genetic association studies of *LXR* genes have been reported in different ethnic populations and diseases, such as obesity [19], metabolic syndrome [20], type 2 diabetes [21-23], Alzheimer’s disease [24,25], inflammatory bowel disease [26] and gallstone disease [27]. Most studies have focused on the *NR1H2* gene and most studies have suggested that the *LXR* gene polymorphisms are associated with susceptibility and outcome of metabolic diseases.

To investigate the effects of polymorphisms on *NR1H3* expression, we used a functional assay of promoter activity in reporter structures that contained mutant type or polymorphic promoters in the Hep3B cell line and COS-7 cell line. Since the Hep3B cell line originates from human hepatocellular carcinoma and LXR is synthesized in the liver, spleen, kidney and macrophages, it was an appropriate cell line for this study. To replicate the Hep3B cell line results, we also tested the promoter activity in the COS-7 cell line from African green monkey kidney [21]. Additionally, the *NR1H3* transcriptional activity according to the stimulants (LPS, GW3965 and T0901317) was analyzed in Hep3B cells and COS-7 cells.

The promoter reporter structure carrying the -1830 C and -1003 A alleles displayed lower promoter activity than the structure carrying the -1830 T and -1003 G alleles in Hep3B cells and COS-7 cells. Moreover, the luciferase activity of the structure containing -1830 C and -1003 A was less enhanced when compared to that of the structure containing -1830 T and -1003 G in GW3965- and T0901317-treated cells.

The -1830 C allele, which is associated with arthritis, displayed significantly lower promoter activity. The -1003 A allele, which has been linked with oral ulcer, arthritis, anti-dsDNA, CNS involvement and triglyceride, displayed significantly lower promoter activity. These findings suggest that LXR regulates inflammatory signaling in SLE patients. It may be possible that under-expressed *NR1H3* leads to autoantibodies production which is against joints and DNA in SLE patients. Besides, the inflammation may increase levels of triglycerides which is a risk factor for atherosclerosis in lupus patients.

The frequency of arthritis was significantly lower in patients who had haplotype HT1 [TTGG]; however, arthritis was more common in patients with HT3 [TCGG]. The frequency of lymphopenia was significantly lower in patients who had haplotype HT2 [CTGG]. These findings suggest that the disease phenotype is more common in patients with SLE who have minor allele -1830 T > C, -1003 G > A and -115 G > A polymorphisms than in those who have the major homozygous genotype. These phenotype results were consistent with the genotype results.

In particular, the -1830 T > C promoter polymorphism was significantly different in genotype analysis and clinical manifestations. The -1830 T > C polymorphisms were located in the promoter site, which is important for gene expression. Therefore, we focused on the functional effects of -1830 T > C polymorphism. To determine if the genetic variants created a transcription factor binding site, sequences were submitted to the TFSEARCH online program, which revealed that the -1830 T > C polymorphism might be a potential GATA-3 binding motif. GATA-3 belongs to the GATA family of transcription factors [28]. The GATA-3 transcription factor is a GATA binding protein 3 that plays a crucial role in Th1 and Th2 development. Some evidence suggests that the GATA-3 transcription factor is correlated with lupus disease activity [29,30].

We performed an EMSA with nuclear extracts from Hep3B cells using double-stranded oligonucleotide probes that corresponded to the -1830 T > C. This polymorphism affected a transcription factor binding in the promoter region of *NR1H3* because a shifted band was found in the presence of the -1830 T probe in the EMSA results. The transcription factor bound to the -1830 T probe was identified as GATA-3 through a competition assay, because GATA-3 was bound to the -1830 T allele promoter with a higher affinity compared with that of the 1830 C allele promoter. We also performed supershift assays using a commercially available anti-GATA-3 antibody and found that the anti-GATA-3 antibody could affect DNA-protein complex formation in the -1830 T probe. The -1830 T specific band was also competed by anti-GATA-3 antibody. Our data suggest that, in the transcription regulation of *NR1H3* gene, GATA-3 might act as a positive regulator because the -1830 T allele containing the reporter construct showed higher promoter activity than the -1830 C allele containing the reporter construct. The results of the present study indicate that the binding affinity of GATA-3 may be decreased in SLE patients who carry the -1830 C allele.

The B cell proliferation assay was performed to determine the effect of -1830 T > C polymorphism. Proliferation of -1830 TC type was increased when compared to that of -1830 TT type in basal, GW3965- and T0901317-treated B cells from SLE patients. Also, proliferation of the -1830 TC type was increased when compared to the -1830 TT type in T0901317-treated B cells from NC. LXR agonist treated cells proliferated less than untreated cells. These findings are consistent with the results of previous studies that have shown that LXR agonist suppresses cell proliferation [31,32].

Moreover, -1830 TC type B cells displayed a lower *NR1H3* mRNA expression level than the -1830 TT type B cells in SLE. Also, the -1830 TC type B cells displayed a lower *NR1H3* mRNA expression level than the -1830 TT type B cells in NC. These mRNA expression and proliferation assay results show that the LXR reduced proliferation of B cells from SLE patients.

In our luciferase assay and proliferation assay, LXR was activated by both T0901317 and GW3965 that has a similar tendency but the T0901317 effect was stronger than that of GW3965. These findings are consistent with the results of previous studies that have shown that activation of PXR targets may explain why T0901317 induces dramatic liver steatosis, while GW3965 has a milder effect [33].

## Conclusions

These results suggest that the *NR1H3* gene genetic polymorphisms may be associated with disease susceptibility and clinical manifestations of SLE. Especially, the -1830 T > C polymorphism within the *NR1H3* promoter region may be involved in regulation of *NR1H3* expression.

## Abbreviations

ACR: American College of Rheumatology; bp: base pair; CI: confidence interval; CNS: central nervous system; co: co-dominant; (D)MEM: (Dulbecco’s) modified Eagle’s medium; do: dominant; DTT: dithiothreitol; EBV: Epstein-Barr virus; EDTA: ethylenediaminetetraactic acid; EMSA: electrophoretic mobility shift assay; FBS: fetal bovine serum; HWE: Hardy-Weinberg equilibrium; Ig: immunoglobulin; IL: interleukin; LPS: lipopolysaccharide; LXR: liver X receptor; MAF: minor allele frequency; NA: not applicable; NC: normal control; OR: odds ratio; PCR: polymerase chain reaction; PMSF: phenylmethanesulfonylfluoride; re: recessive; RT-PCR: reverse transcription PCR; SLE: systemic lupus erythematosus; SNP: single nucleotide polymorphism.

## Competing interests

The authors declare that they have no competing interests.

## Authors’ contributions

JYJ: conception and design, data collection and analysis, manuscript writing and final approval of the manuscript. JYN: data collection and analysis, critical revision and final approval of the manuscript. HAK: data collection and analysis, critical revision and final approval of the manuscript. YBP: data collection and analysis, critical revision and final approval of the manuscript. SCB: data collection and analysis, critical revision and final approval of the manuscript. CHS: conception and design, data collection and analysis, manuscript writing and final approval of the manuscript. All authors read and approved the final manuscript. 

## Supplementary Material

Additional file 1 Clinical characteristics of the study subjects. Click here for file

Additional file 2 **Comparison of the clinical characteristics according to the genotype of *NR1H3 *** **gene in SLE.** Click here for file

Additional file 3 **Comparison of the clinical characteristics according to the haplotype of *NR1H3 *** **gene in SLE.** Click here for file

## References

[B1] Tsokos GC Systemic lupus erythematosus N Engl J Med 2011 365 2110 2121 22129255 10.1056/NEJMra1100359

[B2] D'Cruz DP Khamashta MA Hughes GR Systemic lupus erythematosus Lancet 2007 369 587 596 17307106 10.1016/S0140-6736(07)60279-7

[B3] Suh CH Kim HA Cytokines and their receptors as biomarkers of systemic lupus erythematosus Expert Rev Mol Diagn 2008 8 189 198 18366305 10.1586/14737159.8.2.189

[B4] Rhodes B Vyse TJ The genetics of SLE: an update in the light of genome-wide association studies Rheumatology (Oxford) 2008 47 1603 1611 18611920 10.1093/rheumatology/ken247

[B5] Harley IT Kaufman KM Langefeld CD Harley JB Kelly JA Genetic susceptibility to SLE: new insights from fine mapping and genome-wide association studies Nat Rev Genet 2009 10 285 290 19337289 10.1038/nrg2571 PMC2737697

[B6] Moser KL Kelly JA Lessard CJ Harley JB Recent insights into the genetic basis of systemic lupus erythematosus Genes Immun 2009 10 373 379 19440199 10.1038/gene.2009.39 PMC3144759

[B7] Kaiser R Criswell LA Genetics research in systemic lupus erythematosus for clinicians: methodology, progress, and controversies Curr Opin Rheumatol 2010 22 119 125 20035223 10.1097/BOR.0b013e3283361943

[B8] Rullo OJ Tsao BP Recent insights into the genetic basis of systemic lupus erythematosus Ann Rheum Dis 2012 72 ii56 ii61 23253915 10.1136/annrheumdis-2012-202351 PMC3780983

[B9] Glass CK Witztum JL Atherosclerosis. the road ahead Cell 2001 104 503 516 11239408 10.1016/s0092-8674(01)00238-0

[B10] Joseph SB Bradley MN Castrillo A Bruhn KW Mak PA Pei L Hogenesch J O'Connell RM Cheng G Saez E Miller JF Tontonoz P LXR-dependent gene expression is important for macrophage survival and the innate immune response Cell 2004 119 299 309 15479645 10.1016/j.cell.2004.09.032

[B11] Korf H Vander Beken S Romano M Steffensen KR Stijlemans B Gustafsson JA Grooten J Huygen K Liver X receptors contribute to the protective immune response against Mycobacterium tuberculosis in mice J Clin Invest 2009 119 1626 1637 19436111 10.1172/JCI35288 PMC2689129

[B12] Bensinger SJ Bradley MN Joseph SB Zelcer N Janssen EM Hausner MA Shih R Parks JS Edwards PA Jamieson BD Tontonoz P LXR signaling couples sterol metabolism to proliferation in the acquired immune response Cell 2008 134 97 111 18614014 10.1016/j.cell.2008.04.052 PMC2626438

[B13] Cui G Qin X Wu L Zhang Y Sheng X Yu Q Sheng H Xi B Zhang JZ Zang YQ Liver X receptor (LXR) mediates negative regulation of mouse and human Th17 differentiation J Clin Invest 2011 121 658 670 21266776 10.1172/JCI42974 PMC3026720

[B14] Tan EM Cohen AS Fries JF Masi AT McShane DJ Rothfield NF Schaller JG Talal N Winchester RJ The 1982 revised criteria for the classification of systemic lupus erythematosus Arthritis Rheum 1982 25 1271 1277 7138600 10.1002/art.1780251101

[B15] Jeon JY Kim HA Kim SH Park HS Suh CH Interleukin 6 gene polymorphisms are associated with systemic lupus erythematosus in Koreans J Rheumatol 2010 37 2251 2258 20843912 10.3899/jrheum.100170

[B16] Ogawa D Stone JF Takata Y Blaschke F Chu VH Towler DA Law RE Hsueh WA Bruemmer D Liver x receptor agonists inhibit cytokine-induced osteopontin expression in macrophages through interference with activator protein-1 signaling pathways Circ Res 2005 96 e59 e67 15790955 10.1161/01.RES.0000163630.86796.17

[B17] Barrett JC Fry B Maller J Daly MJ Haploview: analysis and visualization of LD and haplotype maps Bioinformatics 2005 21 263 265 15297300 10.1093/bioinformatics/bth457

[B18] Hedrick PW Gametic disequilibrium measures: proceed with caution Genetics 1987 117 331 341 3666445 10.1093/genetics/117.2.331 PMC1203208

[B19] Dahlman I Nilsson M Jiao H Hoffstedt J Lindgren CM Humphreys K Kere J Gustafsson JA Arner P Dahlman-Wright K Liver X receptor gene polymorphisms and adipose tissue expression levels in obesity Pharmacogenet Genomics 2006 16 881 889 17108812 10.1097/01.fpc.0000236334.49422.48

[B20] Legry V Cottel D Ferrieres J Chinetti G Deroide T Staels B Amouyel P Meirhaeghe A Association between liver X receptor alpha gene polymorphisms and risk of metabolic syndrome in French populations Int J Obes (Lond) 2008 32 421 428 18209740 10.1038/sj.ijo.0803705

[B21] Dahlman I Nilsson M Gu HF Lecoeur C Efendic S Ostenson CG Brismar K Gustafsson JA Froguel P Vaxillaire M Dahlman-Wright K Steffensen KR Functional and genetic analysis in type 2 diabetes of liver X receptor alleles–a cohort study BMC Med Genet 2009 10 27 19292929 10.1186/1471-2350-10-27 PMC2664799

[B22] Solaas K Legry V Retterstol K Berg PR Holven KB Ferrieres J Amouyel P Lien S Romeo J Valtuena J Widhalm K Ruiz JR Dallongeville J Tonstad S Rootwelt H Halvorsen B Nenseter MS Birkeland KI Thorsby PM Meirhaeghe A Nebb HI Suggestive evidence of associations between liver X receptor beta polymorphisms with type 2 diabetes mellitus and obesity in three cohort studies: HUNT2 (Norway), MONICA (France) and HELENA (Europe) BMC Med Genet 2010 11 144 20939869 10.1186/1471-2350-11-144 PMC2958901

[B23] Ketterer C Mussig K Machicao F Stefan N Fritsche A Haring HU Staiger H Genetic variation within the NR1H2 gene encoding liver X receptor beta associates with insulin secretion in subjects at increased risk for type 2 diabetes J Mol Med (Berl) 2011 89 75 81 21042792 10.1007/s00109-010-0687-1

[B24] Adighibe O Arepalli S Duckworth J Hardy J Wavrant-De Vrieze F Genetic variability at the LXR gene (NR1H2) may contribute to the risk of Alzheimer's disease Neurobiol Aging 2006 27 1431 1434 16207502 10.1016/j.neurobiolaging.2005.08.010

[B25] Infante J Rodriguez-Rodriguez E Mateo I Llorca J Vazquez-Higuera JL Berciano J Combarros O Gene-gene interaction between heme oxygenase-1 and liver X receptor-beta and Alzheimer's disease risk Neurobiol Aging 2010 31 710 714 18597895 10.1016/j.neurobiolaging.2008.05.025

[B26] Andersen V Christensen J Ernst A Jacobsen BA Tjonneland A Krarup HB Vogel U Polymorphisms in NF-kappaB, PXR, LXR, PPARgamma and risk of inflammatory bowel disease World J Gastroentrol 2011 17 197 206 10.3748/wjg.v17.i2.197 PMC3020373 21245992

[B27] Schafmayer C Tepel J Franke A Buch S Lieb S Seeger M Lammert F Kremer B Folsch UR Fandrich F Schreiber S Hampe J Investigation of the Lith1 candidate genes ABCB11 and LXRA in human gallstone disease Hepatology 2006 44 650 657 16941683 10.1002/hep.21289

[B28] Zheng W Flavell RA The transcription factor GATA-3 is necessary and sufficient for Th2 cytokine gene expression in CD4 T cells Cell 1997 89 587 596 9160750 10.1016/s0092-8674(00)80240-8

[B29] Chan RW Lai FM Li EK Tam LS Chow KM Li PK Szeto CC Imbalance of Th1/Th2 transcription factors in patients with lupus nephritis Rheumatology (Oxford) 2006 45 951 957 16461436 10.1093/rheumatology/kel029

[B30] Lit LC Wong CK Li EK Tam LS Lam CW Lo YM Elevated gene expression of Th1/Th2 associated transcription factors is correlated with disease activity in patients with systemic lupus erythematosus J Rheumatol 2007 34 89 96 17117487

[B31] Geyeregger R Shehata M Zeyda M Kiefer FW Stuhlmeier KM Porpaczy E Zlabinger GJ Jager U Stulnig TM Liver X receptors interfere with cytokine-induced proliferation and cell survival in normal and leukemic lymphocytes J Leukoc Biol 2009 86 1039 1048 19671841 10.1189/jlb.1008663

[B32] Rough JJ Monroy MA Yerrum S Daly JM Anti-proliferative effect of LXR agonist T0901317 in ovarian carcinoma cells J Ovarian Res 2010 3 13 20504359 10.1186/1757-2215-3-13 PMC2890636

[B33] Mitro N Vargas L Romeo R Koder A Saez E T0901317 is a potent PXR ligand: implications for the biology ascribed to LXR FEBS Lett 2007 581 1721 1726 17418145 10.1016/j.febslet.2007.03.047

