# Supplementary material for: Liver X receptors alpha gene (NR1H3) promoter polymorphisms are associated with systemic lupus erythematosus in Koreans
Source: Arthritis Res Ther. 2014 May 14;16(3):R112. doi: 10.1186/ar4563 (PMC4095571; doi:10.1186/ar4563)
Supplement: Additional file 1 — Clinical characteristics of the study subjects. [file ar4563-S1.doc]

**Additional file 1 Clinical characteristics of the study subjects**

| Characteristics | SLE  n=300 (%) | NC  n=217 (%) | SLE *vs.* NC  *P* value |
| --- | --- | --- | --- |
| Age (year)^*^ | 30.6 ± 9.2 | 29.8 ± 5.8 | 0.240 |
| SEX^*^ Male | 33 (11.0%) | 25 (11.5%) | 0.853 |
| Female | 267 (89.0%) | 192 (88.5%) |
| Oral ulcer^§^ | 148 (49.3%) |  |  |
| Arthritis^§^ | 202 (67.3%) |  |  |
| Serositis^§^ | 40 (13.3%) |  |  |
| Rash^§^ | 117 (39.0%) |  |  |
| Nephritis^§^ | 79 (26.3%) |  |  |
| Leukopenia^§^ | 173 (57.7%) |  |  |
| Lymphopenia^§^ | 272 (90.7%) |  |  |
| Thrombocytopenia^§^ | 46 (15.3%) |  |  |
| Anti-ds DNA antibody ^§^ | 203 (67.7%) |  |  |
| Anti-cardiolipin antibody^§^ | 148 (49.3%) |  |  |
| Lupus anticoagulant^§^ | 57 (19%) |  |  |
| CNS involvement^§^ | 22 (7.3%) |  |  |
| C-reactive protein^*^, mg/dL | 0.12 ± 0.35 |  |  |
| Total cholesterol^*^, mg/dL | 156.2 ± 36.7 |  |  |
| HDL cholesterol^*^, mg/dL | 57.2 ± 17.6 |  |  |
| Triglyceride^*^, mg/dL | 100.9 ± 64.9 |  |  |

^*^ This value was presented as means ± SD. ^§^ This value was presented as number of patients positive for feature or antibody. Clinical manifestation including oral ulcer, arthritis, serositis, rash, nephritis, leukopenia (leukocyte <4×10³ cells/µL), lymphopenia (lymphocyte <1×10³ cells/µL) and thrombocytopenia (thrombocyte <100×10³ cells/µL), anti-dsDNA antibody (>7.0 IU/ml) and anti- cardiolipin antibody (either or both IgG > 20 GPL-U/mL and IgM positive; > 20 MPL-U/mL) were defined by positive involvement when it was positive at least once during the disease duration.
