# Supplementary material for: Liver X receptors alpha gene (NR1H3) promoter polymorphisms are associated with systemic lupus erythematosus in Koreans
Source: Arthritis Res Ther. 2014 May 14;16(3):R112. doi: 10.1186/ar4563 (PMC4095571; doi:10.1186/ar4563)
Supplement: Additional file 2 — Comparison of the clinical characteristics according to the genotype of NR1H3 gene in SLE. [file ar4563-S2.doc]

**Additional file 2 Comparison of the clinical characteristics according to the genotype of *NR1H3* gene in SLE**

| Characteristics | -1830T>C | | | -1003G>A | | | -115G>A | | |
| --- | --- | --- | --- | --- | --- | --- | --- | --- | --- |
|  | TT | CT,CC | *p* | GG | GA,AA | *p* | GG | GA,AA | *p* |
|  | n=238 (79.3%) | n=62 (20.7%) | value | n=250 (83.3%) | n=50 (6.7%) | value | n=235 (78.3%) | n=65 (21.7%) | value |
| Oral ulcer^§^ | 122 (51.3%) | 26 (41.9%) | 0.191 | 130 (52.0%) | 18 (36.0%) | 0.039 | 124 (52.8%) | 24 (36.9%) | 0.024 |
| Arthritis^§^ | 151 (63.4%) | 51 (82.3%) | 0.005 | 160 (64.0%) | 42 (84.0%) | 0.006 | 146 (62.1%) | 56 (86.2%) | <0.001 |
| Serositis^§^ | 30 (12.6%) | 10 (16.1%) | 0.467 | 32 (12.8%) | 8 (16.0%) | 0.543 | 28 (11.9%) | 12 (18.5%) | 0.169 |
| Rash^§^ | 94 (39.5%) | 23 (37.1%) | 0.730 | 94 (37.6%) | 23 (46.0%) | 0.266 | 87 (37.0%) | 30 (46.2%) | 0.182 |
| Nephriti^§^s | 61 (25.6%) | 18 (29.0% | 0.588 | 65 (26.0%) | 14 (28.0%) | 0.769 | 58 (24.7%) | 21 (32.3%) | 0.217 |
| Leukopenia^§^ | 140 (58.8%) | 33 (53.2%) | 0.427 | 143 (57.2%) | 30 (60.0%) | 0.715 | 139 (59.1%) | 34 (52.3%) | 0.323 |
| Lymphopenia^§^ | 216 (90.8%) | 56 (90.3%) | 0.917 | 225 (90.0%) | 47 (94.0%) | 0.375 | 211 (89.8%) | 61 (93.8%) | 0.319 |
| Thrombocytopenia^§^ | 36 (15.1%) | 10 (16.1%) | 0.845 | 38 (15.2%) | 8 (16.0%) | 0.886 | 36 (15.3%) | 10 (15.4%) | 0.990 |
| Anti-ds DNA antibody ^§^ | 156 (65.5%) | 47 (75.8%) | 0.124 | 163 (65.2%) | 40 (80.0%) | 0.041 | 153 (65.1%) | 50 (76.9%) | 0.071 |
| Anti-cardiolipin antibody^§^ | 117 (49.2%) | 31 (50.0%) | 0.735 | 122 (48.8%) | 26 (52.0%) | 0.917 | 114 (48.5%) | 347 (52.3%) | 0.863 |
| Lupus anticoagulant^§^ | 47 (19.7%) | 10 (16.1%) | 0.569 | 46 (18.4%) | 11 (22.0%) | 0.590 | 43 (18.3%) | 14 (21.5%) | 0.345 |
| CNS involvement^§^ | 19 (8.0%) | 3 (4.8%) | 0.295 | 22 (8.8%) | 0 (0.0%) | 0.015 | 20 (8.5%) | 2 (3.1%) | 0.106 |
| C-reactive protein^*^ | 0.12 ± 0.35 | 0.12 ± 0.32 | 0.917 | 0.12 ±0.35 | 0.10 ± 0.33 | 0.700 | 0.12 ± 0.35 | 0.12 ± 0.31 | 0.963 |
| Total cholesterol^*^ | 156.8 ± 37.1 | 154.2 ± 34.9 | 0.630 | 157.0 ± 37.6 | 152.4 ± 31.5 | 0.422 | 157.5 ± 37.4 | 151.9 ± 33.6 | 0.280 |
| HDL cholesterol^*^ | 57.4 ± 18.3 | 56.5 ± 15.2 | 0.886 | 57.6 ± 18.1 | 55.3 ± 15.3 | 0.393 | 57.6 ± 17.7 | 55.7 ± 17.6 | 0.451 |
| Triglyceride^*^ | 97.8 ± 64.1 | 112.8 ± 67.1 | 0.108 | 95.6 ± 61.5 | 126.9 ± 74.9 | 0.007 | 95.3 ± 62.1 | 120.9 ± 71.2 | 0.011 |

**^§^**This value was presented as number of patients positive for feature or antibody. ^*^ This value was presented as means ± SD. Logistic regression analysis was applied to control for age and sex as covariable. No association with SLE phenotypes was observed when the other SNPs were evaluated.
