# Supplementary material for: Liver X receptors alpha gene (NR1H3) promoter polymorphisms are associated with systemic lupus erythematosus in Koreans
Source: Arthritis Res Ther. 2014 May 14;16(3):R112. doi: 10.1186/ar4563 (PMC4095571; doi:10.1186/ar4563)
Supplement: Additional file 3 — Comparison of the clinical characteristics according to the haplotype of NR1H3 gene in SLE. [file ar4563-S3.doc]

**Additional file 3 Comparison of the clinical characteristics according to the haplotype of *NR1H3* gene in SLE**

| Characteristics | HT1 [TTGG] | | | HT2 [CTGG] | | | HT3 [TCGG] | | |
| --- | --- | --- | --- | --- | --- | --- | --- | --- | --- |
|  | +/+ | +/-, -/- | *p* | +/+ | +/-, -/- | *p* | +/+, +/- | -/- | *p* |
|  | n=109 (36.6%) | n=191 (63.4%) | value | n=16 (5.3%) | n=284 (94.7%) | value | n=32 (10.7%) | n=268 (89.8%) | value |
| Oral ulcer^§^ | 53 (48.6%) | 95 (49.7%) | 0.853 | 7 (43.8%) | 141 (49.6%) | 0.646 | 12 (37.5%) | 136 (50.7%) | 0.157 |
| Arthritis^§^ | 62 (56.9%) | 140 (73.3%) | 0.004 | 9 (56.3%) | 193 (68.0%) | 0.331 | 29 (90.6%) | 173 (64.6%) | 0.003 |
| Serositis^§^ | 16 (14.7%) | 24 (12.6%) | 0.605 | 1 (6.3%) | 39 (13.7%) | 0.392 | 4 (12.5%) | 36 (13.4%) | 0.883 |
| Rash^§^ | 45 (41.3%) | 72 (37.7%) | 0.540 | 6 (37.5%) | 111 (39.1%) | 0.899 | 14 (43.8%) | 103 (38.4%) | 0.560 |
| Nephritis^§^ | 33 (30.3%) | 46 (24.1%) | 0.242 | 4 (25.0%) | 75 (26.4%) | 0.901 | 9 (28.1%) | 70 (26.1%) | 0.808 |
| Leukopenia^§^ | 61 (56.0%) | 112 (58.6%) | 0.652 | 11 (68.8%) | 162 (57.0%) | 0.356 | 20 (62.5%) | 153 (57.1%) | 0.558 |
| Lymphopenia^§^ | 100 (91.7%) | 172 (90.1%) | 0.628 | 12 (75.0%) | 260 (91.5%) | 0.027 | 30 (93.8%) | 242 (90.3%) | 0.526 |
| Thrombocytopenia^§^ | 21 (19.3%) | 25 (13.1%) | 0.153 | 1 (6.3%) | 45 (15.8%) | 0.300 | 5 (15.6%) | 41 (15.3%) | 0.961 |
| Anti-ds DNA antibody ^§^ | 74 (67.9%) | 129 (67.5%) | 0.950 | 9 (56.3%) | 194 (68.3%) | 0.316 | 25 (78.1%) | 178 (66.4%) | 0.181 |
| Anti-cardiolipin antibody^§^ | 56 (51.4%) | 92 (48.2%) | 0.698 | 6 (37.5%) | 142 (50.0%) | 0.334 | 15 (46.9%) | 133 (49.6%) | 0.891 |
| Lupus anticoagulant^§^ | 23 (21.1%) | 34 (17.8%) | 0.766 | 3 (18.8%) | 54 (19.0%) | 0.672 | 7 (21.9%) | 50 (18.7%) | 0.902 |
| CNS involvement ^§^ | 9 (8.3%) | 13 (6.8%) | 0.401 | 3 (18.8%) | 19 (6.7%) | 0.103 | 0 (0.0%) | 22 (8.2%) | 0.076 |
| C-reactive protein^*^ | 0.10 ± 0.27 | 0.13 ± 0.39 | 0.478 | 0.18 ±0.58 | 0.12 ± 0.33 | 0.442 | 0.02 ± 0.00 | 0.12 ± 0.35 | 0.678 |
| Total cholesterol | 156.4 ± 37.2 | 156.2 ± 36.5 | 0.968 | 151.0 ± 33.7 | 156.5 ± 36.9 | 0.557 | 172.5 ± 19.1 | 156.1 ± 36.7 | 0.530 |
| HDL cholesterol^*^ | 57.8 ± 19.9 | 56.9 ± 16.3 | 0.660 | 53.5 ± 18.6 | 57.4 ± 17.6 | 0.388 | 63.0 ± 5.7 | 57.2 ± 17.7 | 0.642 |
| Triglyceride^*^ | 96.2 ± 61.7 | 103.6 ± 66.7 | 0.351 | 125.6 ± 109.5 | 99.5 ± 61.4 | 0.117 | 162.0 ± 130.1 | 156.1 ± 36.7 | 0.182 |

**^§^**This value was presented as number of patients positive for feature or antibody. ^*^ This value was presented as means ± SD. Logistic regression analysis was applied to control for age and sex as covariable. No association with SLE phenotypes was observed when the other haplotypes were evaluated. HT: Haplotype.
